# Supplementary material for: Primary Axillary Actinomycosis: A Case Report on the Integration of Culture and Molecular Diagnostics for Accurate Diagnosis of Polymicrobial Infections
Source: Microorganisms. 2025 Mar 17;13(3):671. doi: 10.3390/microorganisms13030671 (PMC11946627; doi:10.3390/microorganisms13030671)
Supplement: Supplementary file 1 [file microorganisms-13-00671-s001.zip › microorganisms-3531491-supplementary.pdf]

## Supplementary Table S1.

PCR Product from FFPE-PCR Performed on Pathological Tissue of the Axilla

| Item | Details |
|---|---|
| Sample source | FFPE-PCR product from pathological tissue of the axilla |
| Identified organism | *Actinomyces gerencseriae* strain KCOM 3578 (=JS484) |
| Genetic target | *16S ribosomal RNA* gene (partial sequence, ~675 bp) |
| Sequence identity | 99.37% (BLAST analysis) |
| Query coverage | 97% |
| GenBank accession number | MW541884.1 |

**Partial Sequence:**

GCCGCACGACTGCTACGAGCCATTGTAGCATGCGTGAAGCCCAAGACATAAGGGGC
ATGATGATTTGACGTCATCCCCACCCTCCTCCGAGTTAACCCCGGCAGTCTCCCGCG
AGTCCCCACCACAACGTGCTGGCAACACGGGACAAGGGTTGCGCTCGTTGCGGGAC
TTAACCCAACATCTCACGACACGAGCTGACGACAACCATGCACCACCTGTGAACCG
ACCCCCACAAAGGGAGGACCCCCCGTCTCCGAAGGACAACCGGCACATGTCAAGCC
TTGGTAAGGTTCTTCGCGTTGCATCGAATTAATCCGCATGCTCCGCCGCTTGTGCGG
GCCCCCGTCAATTCCTTTGAGTTTTAGCCTTGCGGCCGTACTCCCCAGGCGGGGCA
CTTAATGCGTTAGCTACGGCGCGGAAGACCCGGAAAGACCCCCCACACCTAGTGCC
CAACGTTTACAGCGTGGACTACCAGGGTATCTAATCCTGTTCGCTCCCCACGCTTTC
GCTCCTCAGCGTCAGTAACGGCCCAGAGACCCGCCTTCGCCACCGGTGTTCCTCCT
GATATCTGCGCATTCCACCGCTACACCAGGAGTTCCAGCCTCCCCTACCGCACTCAA
GCCAGCCCGTACCCCCCGCAAAGCCATTT

**Reference:**

National Center for Biotechnology Information (NCBI). Basic Local Alignment Search Tool (BLAST). Accessed on March 13, 2025. Available online: https://blast.ncbi.nlm.nih.gov/Blast.cgi
